# Effectiveness of testing, contact tracing and isolation interventions among the general population on reducing transmission of SARS-CoV-2: a systematic review

**DOI:** 10.1098/rsta.2023.0131

**Published:** 2023-10-09

**Authors:** Hannah Littlecott, Clare Herd, John O'Rourke, Lina Toncon Chaparro, Matt Keeling, G. James Rubin, Elizabeth Fearon

**Affiliations:** ^1^ Institute for Medical Information Processing, Biometry and Epidemiology—IBE, Chair of Public Health and Health Services Research, LMU Munich, Germany; ^2^ Institute for Global Health, Faculty of Population Health Sciences, University College London, London, UK; ^3^ Zeeman Institute (SBIDER), Mathematics Institute and School of Life Sciences, University of Warwick, Coventry, UK; ^4^ JUNIPER consortium, UK; ^5^ Department of Psychological Medicine, King's College London, London, UK; ^6^ Department of Global Health and Development, London School of Hygiene and Tropical Medicine, London, UK

**Keywords:** Infectious diseases, SARS-CoV-2, Epidemics, Transmission contol, Testing, Contact tracing

## Abstract

We conducted a systematic literature review of general population testing, contact tracing, case isolation and contact quarantine interventions to assess their effectiveness in reducing SARS-CoV-2 transmission, as implemented in real-world settings. We designed a broad search strategy and aimed to identify peer-reviewed studies of any design provided there was a quantitative measure of effectiveness on a transmission outcome. Studies that assessed the effect of testing or diagnosis on disease outcomes via treatment, but did not assess a transmission outcome, were not included. We focused on interventions implemented among the general population rather than in specific settings; these were from anywhere in the world and published any time after 1 January 2020 until the end of 2022. From 26 720 titles and abstracts, 1181 were reviewed as full text, and 25 met our inclusion criteria. These 25 studies included one randomized control trial (RCT) and the remaining 24 analysed empirical data and made some attempt to control for confounding. Studies included were categorized by the type of intervention: contact tracing (seven studies); specific testing strategies (12 studies); strategies for isolating cases/contacts (four studies); and ‘test, trace, isolate' (TTI) as a part of a package of interventions (two studies). None of the 25 studies were rated at low risk of bias and many were rated as serious risk of bias, particularly due to the likely presence of uncontrolled confounding factors, which was a major challenge in assessing the independent effects of TTI in observational studies. These confounding factors are to be expected from observational studies during an on-going pandemic, when the emphasis was on reducing the epidemic burden rather than trial design. Findings from these 25 studies suggested an important public health role for testing followed by isolation, especially where mass and serial testing was used to reduce transmission. Some of the most compelling analyses came from examining fine-grained within-country data on contact tracing; while broader studies which compared behaviour between countries also often found TTI led to reduced transmission and mortality, this was not universal. There was limited evidence for the benefit of isolation of cases/contacts away from the home environment. One study, an RCT, showed that daily testing of contacts could be a viable strategy to replace lengthy quarantine of contacts. Based on the scarcity of robust empirical evidence, we were not able to draw any firm quantitative conclusions about the quantitative impact of TTI interventions in different epidemic contexts. While the majority of studies found that testing, tracing and isolation reduced transmission, evidence for the scale of this impact is only available for specific scenarios and hence is not necessarily generalizable. Our review therefore emphasizes the need to conduct robust experimental studies that help inform the likely quantitative impact of different TTI interventions on transmission and their optimal design. Work is needed to support such studies in the context of future emerging epidemics, along with assessments of the cost-effectiveness of TTI interventions, which was beyond the scope of this review but will be critical to decision-making.

This article is part of the theme issue ‘The effectiveness of non-pharmaceutical interventions on the COVID-19 pandemic: the evidence’.

## 1. Introduction

Preventing people who have an infectious disease from making contact with others has been one of the cornerstones of public health practice since antiquity [1]. During the COVID-19 pandemic substantial efforts were made by many countries to identify people who were potentially infectious through the use of polymerase chain reaction (PCR) tests or lateral flow viral antigen tests, to isolate these people from others, and to trace and quarantine their contacts—who were also potentially infected. In theory, test, trace and isolate (TTI) methods can work well when the incidence of disease is low, and the generation time is long [2]. The low level of infection means that limited public health resources can be targeted to where they are most needed, and the long generation time means that isolation of the infected individual can occur before many secondary infections are generated. In addition, contact tracing—finding likely secondary infections due to their contact with the identified case—may help to more rapidly identify and prevent further onward transmission from these new infections and further target public health resources. Such testing has been common within public health responses to sexually transmitted infections [3], which often conform to the ideal of low incidence, slow transmission and ease of identifying sexual contacts (although the recent outbreak of Mpox is a counterexample due to the difficulty of identifying the sexual contacts of some of those who were affected [4]).

Testing followed by isolation of the identified infected individual has the potential to break chains of transmission and limit the spread of SARS-CoV-2; when coupled with contact tracing to form TTI there is the potential to accelerate this process. Compared to interventions that aim to reduce transmission through blanket reductions to social contacts, such as stay-at-home orders or workplace and educational closures, TTI interventions aim to identify and prevent contacts between infectious and susceptible people only. They can therefore be seen as more specific strategies compared to population wide social contact reductions, but which could come at a cost to sensitivity and thus epidemic control, if identifying and preventing these contacts is incomplete or delayed. However, as with many public health interventions, there can be a complex range of practical and contextual issues that may affect the strength of the effect. These can be broadly broken down into three categories that relate to: (i) the specific nature of the TTI practices that are employed, (ii) how members of the public engage with these practices and (iii) the current status of the pandemic. In the first category, we include factors such as the speed and sensitivity of the testing mechanism, as well as the speed and accuracy of any contact tracing. The second category includes the public's ability and willingness to follow testing guidance (e.g. testing on early symptoms or testing before entering specific settings) and the extent to which the population is able to adhere to isolation rules [5]. The third category includes the prevalence of infection and the transmissibility of the specific variants that are circulating, as well as the presence of other non-pharmaceutical interventions (NPIs) that reduce the risk of transmission.

### (a) Uses of testing, tracing and isolating

**Figure 1.**
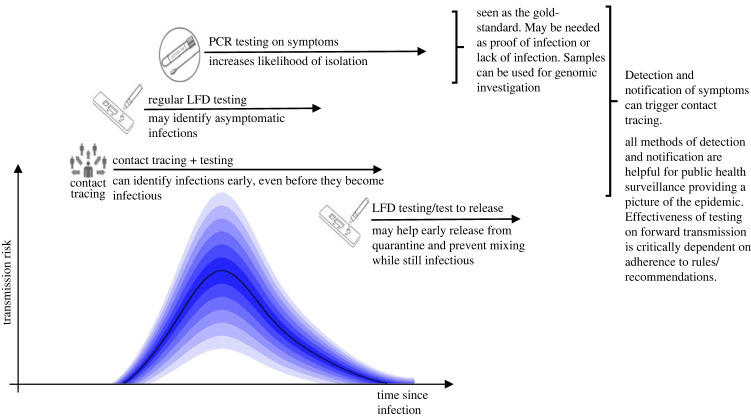
Infographic of the timing and variability in transmission risk from an infected individual with the time since infection, showing the potential timing of symptomatic testing, regular testing, testing following contact tracing and test to release. Early tests combined with isolation were hypothesized to truncate the risk of onward transmission. LFD, lateral flow device; PCR, polymerase chain reaction.

During the COVID-19 pandemic, testing and/or TTI were used in a complex and overlapping range of situations (figure 1), which changed as the epidemic progressed:

1) *Focused testing of symptomatic and high-risk individuals* especially during the early phase of an in-country outbreak, when infection is rare, to contain the spread and prevent transmission among the wider population. This was the case in the UK during the early outbreak, when symptomatic individuals, in combination with other specific travel histories and contact-based criteria, were tested and manual contact tracing performed [6]. In some countries, an intensive TTI system was implemented that aimed to fully contain re-introduced epidemics following periods of zero or very low transmission.
2) *Mass testing of those with symptomatic respiratory disease* to identify SARS-CoV-2 infection, such that individuals could take appropriate action including home isolation and authorities could organize contact tracing. In the UK, this was deployed from May 2020 to April 2022, with minor differences across the four nations [7]; prior to an increase in testing capacity, the UK had adopted a less specific approach of asking everyone with a new onset cough or fever to isolate [8].
3) *Mass testing of those entering healthcare or other vulnerable settings* such as care homes [9]. Here, the aim was to block the interaction between vulnerable susceptible people and those who were infectious, thereby reducing transmission to individuals most likely to suffer severe consequences.
4) *Mass asymptomatic testing of all individuals*, in an attempt to identify both symptomatic and asymptomatic infections and hence dramatically reduce the amount of circulating infection [10].
5) *Regular asymptomatic testing to identify all infectious individuals in workplace and educational settings*. In the UK, this process required healthcare workers [11], care home workers [9,12] and secondary school children [13] to perform regular (twice weekly) tests using lateral flow devices (LFDs) in attempts to minimize spread.
6) *The requirement of a negative test result to undertake particular activities or enter particular settings* [14,15]. Of these, the need for negative tests before international travel was common [16–18].
7) *Testing of identified at-risk individuals*. While the previous testing protocols are concerned with identifying infections, tests could also be used to reduce the time spent in isolation; either to inform the safe early release from isolation [19] or testing at-risk contacts rather than asking them to isolate [20]. In January 2022, the UK advised that people could end self-isolation after 5 days, if they had two negative lateral flow tests taken on consecutive days [21].

Two main forms of diagnostic test were used throughout the COVID-19 pandemic: LFDs and PCR tests [22,23]. PCR and LFD have different targets for detecting SARS-CoV-2: PCR is a laboratory-based system that amplifies and detects viral RNA; LFDs detect the viral antigen [24]. Extensive studies have shown that in general, PCR tests have a higher sensitivity at lower viral loads, thus detecting infections early, as well as beyond the period of high infectiousness, while LFDs are most sensitive when sampled viral load is highest, which is at times of high transmission risk [25–27]. While LFDs have lower sensitivity over the full course of infection, they have the substantial advantage that they provide a rapid assessment (within 15–30 min) that can be performed in the home environment. Here, we do not focus on the differences between PCR- and LFD-based testing, which will be context-dependent, but instead consider the impact that all forms of testing may have on reducing transmission.

When investigating the impact of testing, it is important to consider the wider context of the outbreak, including the presence of other control measures, the incidence of infection and the characteristics of the dominant variant. Control interventions such as mask-use, social distancing or other NPIs aim to reduce the level of transmission from infectious to susceptible individuals, while vaccination aims to reduce the number of susceptible individuals in the population. All of these control measures might help to reduce transmission, such that that TTI can be less effective and still maintain containment or control. PCR testing and manual contact-tracing are highly intensive activities, such that when there is a high incidence of infection resources can become saturated leading to longer delay, weakening the impact of TTI [28]. As the dominant global variants have evolved from wild-type to Alpha, Delta and Omicron there has been an increase in transmission—implying that a greater number of secondary cases need to be prevented to contain the infection. The Omicron variants also have a noticeably shorter generation time [29], meaning that testing, isolation and tracing also need to be achieved more rapidly to maintain their effectiveness.

Another key challenge to assessment of TTI interventions is that the intervention is often also what is used to measure the outcome, e.g. reported confirmed cases. This presents a challenge to evaluation, because a short-term rise in infections detected post-implementation of a TTI intervention could indicate a lack of effectiveness or even harmful effect of the intervention, or it could simply reflect TTI doing what it is intended to do in improving detection of cases.

### (b) Testing and tracing policies

While there have been many different testing policies and practices in different countries [30] and over the course of the pandemic (as knowledge and resources have changed), the impact of any testing policy is fundamentally reliant on human behaviour [31], which might change over time [32], and the extent to which the population is supported to take up and adhere to policies [30,33,34]. Even the most theoretically rigorous TTI intervention is bound to fail if members of the public do not engage with it. For example, if accessing a testing site requires the use of public transport or if information about testing policies is not available in a suitable range of languages, adherence might be lower [35]. Within the UK, concern specifically focused on whether people received adequate financial compensation to enable them to take time off work in order to isolate or to care for children who needed to isolate [36] and whether people recognized that their symptoms necessitated taking a test [37].

Contract tracing policies may also be designed in different ways. In the UK, the National Health Service (NHS) Test and Trace employed a ‘forwards tracing' approach that sought to identify and quarantine people who might have been exposed to a confirmed case during a time when that case would have been infectious. For SARS-CoV-2, this ‘window' was often defined as 2 days before symptom onset (or test date if asymptomatic) and 14 days after, based on what was understood about the duration of infectiousness and timing in relation to symptom onset. A ‘backwards tracing' approach seeks to also identify the source of a confirmed case's infection, which would then enable forwards tracing of additional chains of transmission that might have otherwise been missed. Theoretical modelling work showed that this approach could be particularly effective for an infection like SARS-CoV-2, which was observed to have a highly skewed secondary case distribution—that is, a small proportion of cases led to a disproportionately large number of secondary cases, while many cases led to none or few. Backwards tracing could help to identify those relatively few cases who had transmitted to a high number of secondary transmissions [38,39]. However, there are challenges that might limit effectiveness in practice, such as recall of contacts further back in time [40] and the resources required to implement the approach.

The probability of identifying cases by forward tracing and quarantining them before they become infectious could be further enhanced by tracing and quarantining contacts-of-contacts or secondary contacts, as was implemented in Vietnam [41]. While theoretically more effective at reducing transmission, this approach to tracing and quarantining becomes less specific, and could become so extensive throughout the contact network as to lead to effective ‘mini lockdowns' within local communities, diluting the targeted nature of the isolation measures [42].

Another approach to contact tracing which is similar in practice to backwards and contacts-of-contacts tracing was based on setting. Rather than attempting to trace individual contact links which might be too numerous or difficult to individually determine, cases were instead linked to settings they had attended for a sufficient period during the time that they had been infectious. Contacts were considered to be all other people present at this time and place. While this approach varied over time and was not extended to all types of settings in the UK—not least because of practicality and privacy concerns—at some points during the pandemic in the UK, whole school classes were quarantined without attempting to trace children's individual contacts [43].

One fundamental difference between the COVID-19 pandemic and most previous outbreaks has been the wide-spread use of electronic apps to automate contact tracing [44,45], speeding-up the process, eliminating delays occurring during manual contact tracing and potentially identifying individuals that would otherwise be hard to trace. Most contact tracing apps operate through mobile phones by monitoring local Bluetooth signals. This allows them to record the presence of other phones in the vicinity and hence log ‘close' interactions (e.g. interactions lasting at least 15 min within 2 m). This log of close interactions is only transferred to a centralized system if the phone owner reports a positive test—helping to maintain privacy and security. Phones listed in the log can then be contacted and their users advised to take appropriate action—usually precautionary isolation and testing.

Although testing is primarily a tool for identifying and then isolating infectious individuals, the testing data is also extremely useful for public health monitoring of the outbreak, and hence projection of the likely short- and medium-term behaviour. These analyses and projections can then help a range of government agencies, including health care providers, to plan for future changes in incidence [46]. Additionally, samples sent to laboratories for PCR testing can also undergo genomic sequencing allowing the rapid identification of new variants of concern and highlighting growing risks to the appropriate government agencies.

Here, we have conducted a thorough literature review to identify peer-reviewed publications assessing the real-world effectiveness of testing, contract tracing and isolation/quarantine interventions on the transmission of SARS-CoV-2 infection. Previous reviews of contact tracing for infectious diseases more broadly have included relatively little evidence related to COVID-19 specifically and/or have not included studies looking only at testing and/or isolation intervention components [47,48]. The evidence presented in this article has accumulated over the course of the COVID-19 pandemic; it was not present at the start of the outbreak to directly inform policy makers. Similarly, data specific to any future outbreak will take time to generate, although some of the general principles shown here are likely to hold.

## 2. Methods

This review focused on studies that quantitatively assessed the real-world impact on SARS-CoV-2 transmission outcomes of testing, contact tracing and isolation/quarantine interventions during the COVID-19 pandemic. The review forms one part of a larger project focusing on a range of non-pharmaceutical measures to contain the COVID-19 pandemic, funded by the Royal Society. To enhance the quality of this rapid review, we followed the Cochrane rapid review guidance [49], with a modification noted below.

### (a) Inclusion and exclusion criteria

We included studies that provided a quantitative measure of real-world TTI effectiveness on reducing SARS-CoV-2 transmission. These included but were not restricted to the following study designs: randomized controlled studies (RCTs); prospective cohort studies; retrospective cohort studies; controlled before-and-after studies; interrupted time series; case-control studies; cross-sectional studies; and mixed methods studies (if a quantitative measure of impact could be extracted). Concentrating on real-world effectiveness studies, we excluded mathematical modelling studies; however, we did identify them during abstract and full paper screening and so are able to provide a list of mathematical modelling studies otherwise fitting our inclusion criteria as ‘supporting studies', listed in electronic supplementary material, appendix 3. For observational studies, we anticipated that many measured and unmeasured confounding factors could be present, including but not limited to differences in population demographics, immunity, infection variants, other transmission control interventions in place at the time, epidemic dynamics and test-seeking behaviour (assuming this is not an intervention under study). We therefore restricted full extraction and inclusion to studies that attempted to adjust for such confounding factors. Potential for risk of bias due to confounding was still assessed as part of study methodological appraisal, as described in more detail below. Uncontrolled observational studies were listed as supporting studies in electronic supplementary material, appendix 3.

We included only studies published in peer-reviewed journals which provides an additional element of quality control to our review; studies published only on preprint servers were excluded. Also, the following types of study were excluded: studies with no quantitative measure of impact; purely qualitative studies; diagnostic studies focusing solely on sensitivity and specificity of testing; non-empirical studies (e.g. commentaries, editorials, literature reviews); systematic reviews; and conference abstracts or reports.

We included studies assessing transmission and TTI effectiveness in the general population only. This general population could occur in any global location, with no restrictions relating to age, gender or health conditions. Any studies in which interventions were delivered in specific settings only were excluded. These included, but were not limited to: care homes; hospitals and other healthcare settings; schools; specific workplace types; and prisons. Given the high degree of vulnerability in some of these settings and the degree of close clustered contact, results in these settings may amplify the impacts of controls meaning that their findings might not be applicable outside the setting.

Studies were included if they focused on each of the following four components, in combination with each other or individually:

1. SARS CoV-2 testing in combination with a transmission reduction element based on the results of the test, including: isolation initiation; contact tracing; prevention from attending setting (e.g. care home, workplace, school, healthcare setting); prevention from attending events; and release from isolation. Studies focusing on diagnostic testing to identify clinical treatment and pathways with no explicit transmission outcome were excluded. This means that we did not include studies whose primary outcome was an assessment of test sensitivity and specificity, which did not go on to assess the use of these tests in an intervention to reduce transmission.
2. Contact tracing, if this is in combination with transmission reduction elements, including: quarantine initiation; and SARS-CoV-2 testing.
3. Isolation of probable infectious cases (PCR/antigen tested or symptom-based), including interventions to facilitate and enable successful contact reduction while in isolation (e.g. advice, support payments, accommodation provision).
4. Quarantine of exposed contacts of infectious cases, including interventions to facilitate successful contact reduction for the duration of quarantine (e.g. advice, support payments).

Studies of interventions employing TTI components which did not measure transmission outcomes were excluded; this may include interventions aiming to improve treatment outcomes among those infected or studies for which a transmission outcome is not reported. Studies where TTI was employed as part of an international border policy were also excluded from our analysis because these studies were included in a separate review on border policies. To make a meaningful assessment of the impact of TTI components requires a comparator or counterfactual; in our analysis, eligible comparators included populations, settings or time periods with no TTI intervention or a variation in intensity to the intervention under study. Intervention versus non-intervention assessment were included, for instance, the random assignment of individuals to testing or non-testing (control) groups, or the controlled comparison of a time period in which contact tracing was implemented to a time period when it was not. Variation in intervention intensity included examples of differential numbers of tests *per capita* in different regions or changes to contact tracing (as measured by a standardized scale) over time. Although the focus of this review was on the reduction in transmission due to TTI interventions, transmission was rarely measured directly, so other outcomes of interest were assessed. These transmission-related outcomes are listed below:

— positive SARS-CoV-2 tested infections (PCR or LFDs)
— secondary/tertiary attack rates
— estimated incidence
— estimated infections averted
— growth rate of confirmed cases or deaths
— the effective reproductive ratio *R* or *R_t_*
— peak height/incidence per day
— deaths
— rates of hospitalization
— intensive care unit (ICU) bed usage

Deaths, hospitalizations and ICU bed usage outcomes were also included, but only if these were used as indicators of changes in rates of infection rather than changes in rates of treatment conditional on infection.

Inclusion and exclusion criteria are summarized in table 1.

**Table 1.** Review inclusion and exclusion criteria. NGO, non-governmental organization; TTI, test trace isolate.

	included	excluded
population	studies reporting about TTI interventions conducted in the general population	TTI interventions implemented for and within specific settings, such as prisons, care homes, schools, hospitals and healthcare settings, military barracks
intervention	study interventions included: testing in combination with contact tracing and/or isolationorcontact tracing in combination with quarantineorisolation of suspected or confirmed cases (may or may not include testing)	interventions described as ‘quarantine' which were applied to the general population indiscriminately (i.e. not based on symptoms, test results or contact with a confirmed case) border control interventions (i.e. testing and quarantine interventions implemented as pre- or post-international travel requirements)
comparison	groups receiving:— no TTI intervention— less intense TTI intervention— alternative TTI interventionscomparison could be across time, place or population	studies that were descriptive only
outcome	measures of effect on transmission, which could include incidence, numbers of cases, reproduction number, growth rate, deaths (when used to assess transmission), hospitalizations (when used to assess transmission)	measures indicating to treatment success rather than transmission reduction (e.g. deaths or hospitalizations among those with already identified infection) test validation metrics such as sensitivity, specificity, positive or negative predictive value
study design	randomized controlled trials; prospective cohort studies; retrospective cohort studies; controlled before-and-after studies; time series; case-control studies; cross-sectional studies IF there was an attempt to control for confounding factors	Mathematical transmission model Observational study with no attempt to control for confounding
language	studies published in English	studies where the full article was not in English
publication	peer-reviewed journal articles	pre-printsconference abstracts where a full paper was not availablestudy protocols where a paper giving results was not yet availablegrey literature (government reports, NGO reports, working papers)
disease	COVID-19 (with synonyms, see search strategy) caused by SARS-CoV-2 infection	any other disease/infection
dates	all studies meeting other inclusion criteria	none

### (b) Search strategy

We searched the literature using four main search components: SARS-CoV-2/COVID-19; testing; contact tracing; and isolation/quarantine, details of which can be found in electronic supplementary material, appendix 1. Systematic searches were run on the 6 January 2023 in Ovid MEDLINE (1946 to present) electronic databases. Results were restricted to 2020 onwards and limited to English language only. We also searched two COVID-19-specific databases on 9 December 2020. Firstly, the Cochrane COVID-19 Study Register (covid-19.cochrane.org), which contains study references from ClinicalTrials.gov, WHO International Clinical Trials Registry Platform (ICTRP), PubMed, Embase, CENTRAL, medRxiv and other handsearched articles from publishers' websites; and secondly the WHO COVID-19 Research Database [50], restricted to specific primary sources.

### (c) Screening

Search results underwent deduplication using Endnote 20. After this, title and abstract screening was undertaken using Rayyan software [51]. First, title and abstract screening was calibrated, whereby all reviewers screened the same 20 records, before comparing results and clarifying the application of inclusion and exclusion criteria. All titles and abstracts were then screened by at least one reviewer, with 20% being screened by two reviewers (‘dual-screening’) to enhance screening quality. Any conflicts were resolved by a third reviewer. This was a pragmatic modification to the Cochrane Rapid Review guidance that at least 20% of abstracts are dual-screened with a recommendation that a second review is conducted for all excluded abstracts. Of the 20% of dual-screened abstracts, 373 (7% of dual-screened abstracts) were given a second opinion due to low confidence of the initial reviewer as to whether the abstract should be included or excluded and 4868 (93% of dual-screened abstracts) were not individually selected but were assigned in batches prioritizing reviewers with less reviewing and/or TTI research experience, though covering all reviewers.

Full text screening began with calibration, whereby all reviewers screened the same 10 full texts, before discussing and clarifying the application of inclusion and exclusion criteria. All full texts were screened by at least one reviewer, with 317 (27% of all full texts) dual-screened by two reviewers. Any conflicts were resolved by a third reviewer. Full texts for dual screening included those for which the first reviewer had low confidence in their judgement and those which were not individually selected but were drawn in batches disproportionately from reviewers with less experience. Reasons for exclusion were recorded at this stage and full text screening was managed using a shared Excel database.

### (d) Data extraction

The data extraction form was piloted before study characteristics and data were extracted by a single reviewer and checked by a second reviewer. This stage was managed using a shared database. Data extraction consisted of the following categories: study information; methods; population and setting; intervention type; findings; and contextual information. The data extraction form headings can be viewed in electronic supplementary material, appendix 2.

### (e) Risk of bias assessment

Risk of bias assessments were undertaken by one reviewer and all were reviewed by another for consistency. The Cochrane RoB 2 tool [52] was employed for randomized controlled trials. We used ROBINS-I for the assessment of non-randomized studies of interventions [53], guided by the *Cochrane Handbook for Systematic Reviews of Interventions* for assessing risk of bias of different types of non-randomized studies [54]. ROBINS-I requires the identification of potentially important domains of confounding *a priori*, as well as imagining a target trial of best practice for each study to be compared to. These were differences in: sociodemographic characteristics between intervention and comparison groups, epidemic dynamics over the time period of the study, population immunity, dominant SARS-CoV-2 variant and co-occuring interventions during the study period and means of measuring the outcome. Additional domains might be identified in individual studies. Each study was assessed as either ‘low', ‘moderate', ‘serious' or ‘critical' risk of bias. Any study rated at critical risk of bias was excluded from the review, in alignment with Cochrane Handbook guidance. For RoB-2, studies were rated as ‘low' risk, as having ‘some concerns' or as having a ‘high' risk of bias. Risk of bias ratings for each study are summarized in electronic supplementary material, appendix 4.

### (f) Synthesis

Data were narratively synthesized using the synthesis without meta-analysis (SWiM) guidance [55]. Studies were separated according to the intervention, or combination of test, trace and isolate interventions for which they reported data. Studies were included in multiple sub-sections if they reported on more than one intervention. Extracted data were then summarized narratively, and presented in a table.

## 3. Results

**Figure 2.**
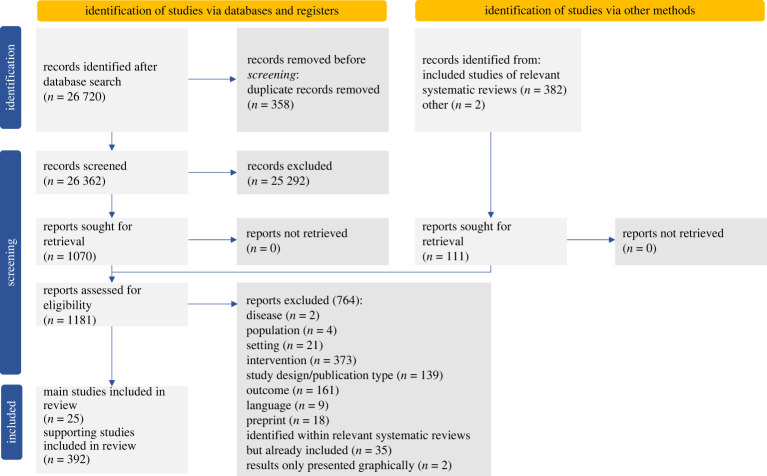
PRISMA flow diagram showing the source of studies identified during screening stages, reasons for exclusion and the final number of included and supporting studies.

The PRISMA flow diagram (figure 2) shows the sources and numbers of articles identified at each stage of screening. The database search identified 26 720 unique records, of which 358 were duplicate records. After screening the titles and abstracts of the remaining 26 362 papers, 1070 were taken forward into full text screening, and an additional 111 studies identified via examining the references of related systematic reviews also screened. From these, we identified 25 studies [10,20,56–78] that met all our inclusion criteria. Reasons for exclusion are included in figure 2. There were two studies which presented results only graphically, and it was not possible to extract quantitative findings [79,80]. There were 374 peer-reviewed studies which were mathematical transmission modelling studies and 18 uncontrolled observational studies, not included in the review but listed in electronic supplementary material, appendix 3.

Many of the 374 transmission model-based studies used compartmental models in which an isolation or quarantine class was added to the standard susceptible infectious recovered (SIR) model paradigm. Transmission models varied in their sophistication and their real-world applicability, with some models focusing on generic concepts of invasion and stability while others more closely matched the dynamics of SARS-CoV-2 spread in their chosen focal areas. The majority of these model-based studies concluded that adding an isolation class (into which infected individuals can be diverted) reduces the transmission of SARS-CoV-2. The strength of this reduction is critically dependent on model assumptions and parameters, which may be difficult to correctly infer without very detailed individual-level data.

### (a) Description of included studies

Included study characteristics are given in table 2. One included study was an individually randomized controlled trial [67]. Eleven studies used observational time-series designs [56,57,60,62,64,70–73,75,78]. These compared variation across, for instance, geographical time units while variously adjusting for characteristics of the units, other interventions and features of the epidemic. Some studies assumed or investigated time lags between exposures and outcomes, assuming that the effects of the intervention would take time to impact transmission. Two studies used difference-in-difference designs [58,76] comparing the change over time in between groups exposed and unexposed to the intervention, accounting for baseline characteristics and thus more robustly controlling for confounding than in observational time-series designs. Three used synthetic control designs [65,69,77], reconstructing a counterfactual ‘unexposed' scenario for the intervention group, based on the trends in unexposed controls [82] and thus enabling better control for time-varying confounding. One study used a controlled before-and-after design [10]. There were two retrospective [59,68] and two prospective cohort designs [20,61] which compared the transmission outcome over time among individuals who were either exposed or unexposed to the TTI intervention of interest. Three studies were cross-sectional and used cumulative outcomes: cases by a given time [74], drop in growth post peak incidence [63] and *per capita* mortality [66].

**Table 2.** Characteristics of the 25 included studies

study	study design	location/ population	sample	dates of data collection	data sources	TTI intervention type(s)	TTI intervention description
contact tracing							
Deng *et al*. [58]. The effectiveness and costs of nonpharmaceutical interventions for COVID-19 containment: a border discontinuous difference-in-difference approach	difference-in-difference	China, general population	84 229 confirmed COVID-19 cases and 78 974 recovered cases in 321 cities	3 Jan to 30 July 2020	laboratory-confirmed cases reported by the health departments of each provincial government	contact tracing	high intensity exposure to contact tracing. Initially manual tracing; digital tracing introduced partway through the study period
Fernandez-Nino*et al*. [59]. Effectiveness of contact tracing to reduce fatality from COVID-19: preliminary evidence from Colombia	retrospective cohort	Colombia, general population	1.4 million cases, 542 936 chains of contact, and 46 087 deaths	28 Mar 2020 to 13 Jan 2021	Government nationwide registry (SEGCOVID19). This system combines information of suspected and confirmed cases reported to the National Public Health Surveillance System (SIVIGILA in Spanish) with their contacts identified through direct tracing. Data on all COVID-19 tests in Colombia from the national sampling system	contact tracing	contact tracing as implemented in the ‘Tests, Tracing and Select Sustainable Isolation Program' (‘PRASS’ in Spanish)
Fetzer *et al*. [76]. Measuring the scientific effectiveness of contact tracing: evidence from a natural experiment	difference-in-difference	England (UK), general population	315 English local authorities. 15 841 positive cases were missed from reporting figures	Sept–Oct 2020	Government reported PCR-positive confirmed cases. Data from NHS Test and Trace (government agency conducting contact tracing)	contact tracing	manual contact tracing as implemented by NHS Test and Trace (household isolation of identified contacts, symptomatic testing only)
Gianino *et al*. [60]. Evaluation of the strategies to control COVID-19 pandemic in four European Countries	time series	Italy, Germany, Spain, United Kingdom. General populations	four countries over 3.5 months	1 Oct 2020 to 10 Jan 2021	OxCGRT	contact tracing	contact tracing as measured by the OxCGRT
Kendall *et al*. [65]. Epidemiological changes on the Isle of Wight after the launch of the NHS Test and Trace programme: a preliminary analysis	synthetic control, and before and after	England	150 Upper Tier Local Authorities	28 Mar 2020 to 14 June 2020	COVID-19 daily case data from Public Health England	contact tracing	combined programme of manual contact tracing and a mobile phone contact tracing app; health advice to symptomatic people and details about testing; health advice to app-identified contacts of cases on reducing transmission (not self-isolation guidance). Note, manual tracing became available everywhere in England through NHS Test and Trace May 28, 2020, so the intervention assessed and the comparison made varies over the study period
Vecino-Ortiz*et al*. [73]. Impact of contact tracing on COVID-19 mortality: an impact evaluation using surveillance data from Colombia	time series	Colombia, general population	54 931 cases occurring in 37 geographical units (32 departments and five districts)	2 Mar to 16 June 2020	data on all PCR-confirmed COVID-19 cases obtained from the ‘Open Data' portal of the Colombian National Institute of Health. Control intervention data from the Google Community Mobility Reports	contact tracing	contact tracing of PCR-confirmed cases and isolation of contacts
Wymant *et al*. [74]. The epidemiological impact of the NHS COVID-19 app	cross-sectional (using cumulative outcomes)	England and Wales (UK), general population	338 Lower Tier Local Authorities in England and Wales (LTLAs)	24 Sept 2020 to 31 Dec 2020	app use data. Case data from Public Health England at https://coronavirus.data.gov.uk/. Data about LTLA characteristics from the Office for National Statistics. Information about manual tracing from NHS Test and Trace data	contact tracing	contact tracing via a mobile app
testing strategies							
Chew *et al*. [57]. Data-driven multiscale modelling and analysis of COVID-19 spatio-temporal evolution using explainable AI	time series	cross-country, general population	greater than 150 countries *(specific number not given)*	2 May 2020 to 1 Oct 2021	Ourworldindata.org, which draws case data from JHU CSSE COVID-19 [81] data and intervention data from OxCGRT	testing strategies	exact intervention variable across countries
Gorji *et al*. [20]. Results from Canton Grisons of Switzerland suggest repetitive testing reduces SARS-CoV-2 incidence (February–March 2021)	prospective cohort	Canton Grisons, Switzerland, labour force	27 514 employees without symptoms from 1022 businesses, entered into the intervention in three weekly cohorts	Feb to Mar 2021	intervention testing data (17% or 25 449/146 826 of individual weekly entries had missing results and were removed from the dataset)	testing strategies	weekly asymptomatic saliva testing followed by self-isolation at home if positive. Contacts of cases daily test on LFD for 10 days; do not isolate while testing negative
Haug *et al*. [64]. Ranking the effectiveness of worldwide COVID-19 government interventions	time series	cross-country, general population	56 countries, 79 territories (data from the US were available at state level)	Mar to Apr 2020	on interventions: Publicly available Complexity Science Hub COVID-19 Control Strategies List dataset on NPIs (Desvars-Larrive, A *et al.* A structured open dataset of government interventions in response to COVID-19. *Sci. Data* 7, 285 (2020)); CoronaNet COVID-19 Government Response Event Dataset (Cheng C, Barceló J, Hartnett AS, Kubinec R, Messerschmidt L. 2020 COVID-19 government response event dataset (CoronaNet v.1.0). *Nat. Hum. Behav.* **4**, 756–768.); WHO-PHSM dataset (Tracking Public Health and Social Measures: A Global Dataset (World Health Organization, 2020); https://www.who.int/emergencies/diseases/novel-coronavirus-2019/phsm). Johns Hopkins Interactive Dashboard [81]	testing strategies	variations in intensity of quarantine and in enhanced case detection. Exact interventions vary by country
Hong *et al*. [63]. Effect of COVID-19 non-pharmaceutical interventions and the implications for human rights	cross-sectional using cumulative outcomes	cross-country, general population	108 countries	1 Jan 2020 to 15 Jun 2020	OxCGRT dataset	testing strategies	effect modification of other epidemic control interventions by contact tracing as measured by the OxCGRT scale
Leffler *et al*. [66]. Association of country-wide coronavirus mortality with demographics, testing, lockdowns, and public wearing of masks	cross-sectional	cross-country, general population	169 countries	Apr 2020	mortality data tabulated by Worldometer, downloaded from the Internet Archive. OxCGRT	testing strategies	testing and contact tracing as measured by the OxCGRT
Pavelka *et al*. [10]. The impact of population-wide rapid antigen testingon SARS-CoV-2 prevalence in Slovakia	before and after	Slovakia, general population (children and older adults excluded from testing intervention)	79 counties with at least 2 rounds of testing	Oct to Nov 2020	testing intervention data. Routine syndromic and PCR confirmed surveillance for the daily incidence of infections as reported by the Slovak Ministry of Health	testing strategies	lockdown exit via synchronised mass population-wide testing with rapid antigen tests and home isolation (10 days isolation following positive test result for the case, household and traced contacts)
Pozo-Martin *et al*. [70]. The impact of non-pharmaceutical interventions on COVID-19 epidemic growth in the 37 OECD member states	time series	cross-country, general population	37 countries, over 11 weeks, total of 407 data points	Jan– July 2020. Model tested against data from Oct to Dec 2020	OxCGRT for both cases and intervention data	testing strategies	testing volumes (as proxy for testing policy) and contact tracing as measured using the OxCGRT
Spiliopoulos [71]. On the effectiveness of COVID-19 restrictions and lockdowns: pan metron ariston	time series	cross-country, general population	132 countries	15 Feb 2020 to 14 Apr 2021	OxCGRT for intervention data. Confirmed case and death data taken from Guidotti E, Ardia D. COVID-19 Data Hub. J Open Source Softw. 2020;5(51):2376. Guidotti E. A worldwide epidemiological database for COVID-19 at fine- grained spatial resolution. *Sci Data*. 2022;9(1):112	testing strategies	contact tracing and testing policies as measured by the OxCGRT
Ssentongo *et al*. [72]. Pan-African evolution of within- and between-country COVID-19 dynamics	time series	African cross-country. general population	46 countries	Feb 2020 to 13 Aug 2020	OxCGRT. Human Development Index. UK Met-Office numerical weather prediction model output (Walters *et al*. The Met Office Unified Model Global Atmosphere 7.0/7.1 and JULESGlobal Land 7.0 configurations. *Geosci. Model Dev.* (GMD) 12, 1909–1963 (2019))	testing strategies	testing policies as measured by the OxCGRT
Yalaman *et al*. [75]. Cross-country evidence on the association between contact tracing and COVID19 case fatality rates	time series	cross-country, general population	138 countries, over 10 months split into 731 two-week time period (634 when excluding outliers)	Jan 2020 to Oct 2020	OxCGRT. Case and death data from ourworldindata.org	testing strategies	testing and contact tracing intensity as measured by the OxCGRT
Zamanzadeh & Cavoli [78]. The effect of nonpharmaceutical interventions on COVID-19 infections for lower and middle-income countries: A debiased LASSO approach	time series	low and middle income countries, general population	37 countries over 100 days	date of first in-country infection, followed by 100 days. Overall13 Jan 2020 to 2 July 2020	OxCGRT for epidemic control measures including contact tracing. Our World in Data for the ration of numbers of infections and numbers of tests per 100 000 population. Country socio-demographic, economic and healthcare indicators from the World Bank and the World Health Organization	contact tracingTesting Strategies	contact tracing policies as measured by the OxCGRT and numbers of tests reported *per capita*
Zhang *et al*. [77]. Impact of community asymptomatic rapid antigen testing on COVID-19 related hospital admissions: synthetic control study	synthetic control	England	61 small geographic areas in Liverpool and 6290 small geographic areas in England but not Liverpool, used to construct the synthetic control	6 Nov 2020 to 2 Jan 2021	National Health Service Hospital Episodes Statistics data. Sociodemographic characteristics of areas informed using government Office for National Statistics data	testing strategies	provision and encouragement for free supervised rapid testing using lateral flow device tests for people without symptoms living or working in the city of Liverpool. Positive tests were followed by 10 days self-isolation at home and a confirmatory PCR test
isolation							
Lopez *et al*. [61]. Impact of isolating COVID-19 patients in a supervised community facility on transmission reduction among household members	prospective cohort	Spain, general population	89 individual cases and their 229 contacts	Apr to June 2020	index patients were identified from the hotel admission lists and primary care electronic medical records. The participant sample was selected consecutively from these lists	isolation strategies	hotel isolation of cases (compared to home isolation)
Love *et al*. [67]. Daily use of lateral flow devices by contacts of confirmed COVID-19 cases to enable exemption from isolation compared with standard self-isolation to reduce onward transmission of SARS-CoV-2 in England: a randomized, controlled, non-inferiority trial	individually randomized control trial. Non-inferiority	England, general population (adults)	number randomised: 28 757 to the intervention; 26 166 to the control. Total: 54 923 contacts of confirmed cases	29 Apr 2021 to 28 July 2021	routinely collected NHS Test and Trace (government) testing data	isolation strategies	daily lateral flow detection tests (LFD tests) for 7 days among contacts of confirmed cases instead of home isolation for 10 days. Home isolation for 10 days if test-positive on LFD during the testing period
Malheiro *et al*. [68]. Effectiveness of contact tracing and quarantine on reducing COVID-19 transmission: a retrospective cohort study	retrospective cohort	Portugal, general population	551 individual cases	1 Mar to 20 Apr 2020	routinely collected case and contact tracing data	isolation strategies	manual contact tracing and mandatory quarantine with daily symptom check calls. Intervention and control cohorts were defined based on whether cases were subjected to contact tracing and quarantine measures before the laboratory confirmation of disease
Nam *et al*. [69]. Early centralized isolation strategy for all confirmed cases of COVID-19 remains a core intervention to disrupt the pandemic spreading significantly	synthetic control	China, Hong Kong, Taiwan, Singapore, Korea, Japan, the United States, France, Germany, the United Kingdom, Canada, Italy, Spain and Sweden. General populations	14 countries	22 Jan 2020 to 31 Mar 2020	data from John Hopkins Interactive Dashboard [81] and WHO Situation Reports. Data also extracted from official governmental announcements, health ministry updates, creditable and verified articles	isolation strategies	mandatory centralized isolation of cases (outside of the home in varying locations)
TTI as part of a package							
Chan *et al*. [56]. COVID-19 non-pharmaceutical intervention portfolio effectiveness and risk communication predominance	time series	cross-country, general population	50 countries	Jan (variable depending on first case identified) to 7 June 2020	Japan: local dataset (Dashboard and Map of COVID-19 Japan Case. https://gis.jag-japan.com/covid19jp/, 2020). Other 49 countries: WHO and John Hopkins dashboard (WHO. Coronavirus disease (COVID-19) Situation Report. https://www.who.int/emergencies/diseases/novel-coronavirus-2019/situation-reports (2020). Johns Hopkins Interactive Dashboard [81]	TTI as part of a broader package of measures	case identification, contact tracing and related measures. Exact interventions variable across countries
Heo *et al*. [62]. Statistical estimation of effects of implemented government policies on COVID-19 situation in South Korea	time series	South Korea, general population	one country over 10 month's follow-up days	20 Jan 2020 to 20 Nov 2020	case data obtained from Kaggle website until 30 June and then used the Korea public data portal of the Ministry of Health and Welfare. Intervention data from OxCGRT	TTI as part of a broader package of measures	average measure of a ‘health’ intervention index comprised of public information campaign, testing policy and contact tracing intensity measured using the OxCGRT scale

Of the 25 studies included, 12 used data from multiple countries across Europe, America, Africa and Asia [56,57,60,63,64,66,69–72,75,78]. From the remaining studies, there were nine studies based in Europe, including five from the UK or England [65,67,74,76,77], and one from each of the following countries: Portugal [68]; Spain [61]; Switzerland [20] and Slovakia [10]. Two studies were based in Asia, one from China [58] and one from South Korea [62] and the final two studies were based in Colombia [59,73].

All but one study examined policies implemented across the general population, while one examined testing conducted among the labour force [20].

The interventions examined in the 25 included studies varied depending on which activities were undertaken during the study period, either testing, tracing and isolation or a combination of the three. TTI intervention exposure measures included continuous measures, such as the number of tests performed *per capita* as well as ordinal response scales of TTI intervention intensity (e.g. the commonly used Oxford COVID-19 Government Response Tracker (OxCGRT) [83] which used a 4-point scale to characterize testing policy and a 3-point scale for contact tracing), finding an effect of a one category increase in intensity. The RCT compared assignment to the intervention arm (daily contact testing) and assignment to the control arm (contact home isolation) [67].

Outcomes were categorized into three groups; transmission, healthcare usage (as a proxy of transmission) and deaths. Within the transmission category, 12 studies focused on number of cases, three focused on reproduction number, three on onward transmission, two on growth rate and one on relative risk. Several studies reported more than one outcome. Six studies measured number of deaths. One study used hospitalizations, over a given time period, as the transmission outcome because hospitalizations are less affected by changes in levels of case detection than other outcomes [77].

One study was assessed using the Cochrane RoB 2 tool for RCTs and the risk of bias was assessed to be as of ‘some concerns’ [67]. The remaining 24 studies were assessed using the ROBINS-I tool for non-randomized study designs; of these, six were assessed as moderate risk of bias [10,58,65,74,76,77] and the remaining 18 were assessed as serious risk of bias. The risk of bias analysis is summarized in electronic supplementary material, appendix 4.

### (b) Intervention effects

Studies were separated into four groups according to the elements of TTI investigated: contact tracing only (seven papers); specific testing strategies, in some cases alongside contact tracing (12 papers); strategies for isolating cases/contacts (four papers); and TTI as part of an intervention package (two papers). The majority of studies use data from 2020, as such the results are largely unaffected by variants, vaccination or population-level immunity.

Detailed quantitative study findings, including analytical methods and statistical uncertainty, are given in table 3.

**Table 3.** Summary findings of the 25 included studies.

study	exposure measure	outcome	analysis method	control of confounding	results
contact tracing					
Deng *et al*. [58]	treatment versus control cities. Treatment cities are defined as cities where the residual of the regression of NPIs stringency on the number of cumulative confirmed cases by 23 January is positive; cities not meeting this criterion were controls	logarithmic cumulative confirmed cases	border discontinuous difference-in-difference (DiD) method, with exogenous variations in the stringency of NPIs to identify the causal effect of NPIs on COVID-19 spread. (Control and treatment cities sharing a border were matched)	weighed all the DiD regressions by performing propensity-score-matching of migrant inflows and characteristics that affect the spread of SARS-CoV-2. Used staked DiD regression analysis to control for other intervention effects. The specifications include city-specific time trends as days since the first confirmed case, and its square term. Cumulative confirmed cases in 7 days leading up to the current date were included to control the loading of local public health systems. Weather controls included the daily average temperature, relative humidity, and wind speed for the 7 days leading up to the current date. All the regressions included city fixed effects and week fixed effects	35.8% reduction in cases in treatment versus control cities (s.e. 6.1%), statistically significant at the 1% level, within 6 days of contact tracing initiation (adjusted). Smaller effects seen over longer time periods from initiation (18.5% reduction (s.e. 1.6%) within 13 days post-initiation; 4.1% reduction (s.e. 1.5%) within 20 days post-initiation, both significant at the 1% level. Effects after 21 days were not statistically significant)
Fernandez-Nino*et al*. [59]	contact tracing chains either do or do not contain five or more contacts per case. Used as an indicator of contact tracing intensity	number of COVID-19 deaths in each contact tracing chain, (chain analysis). Death from COVID-19 (individual level analysis)	multilevel negative binomial model with the number of people within the chain as an offset, (chain analysis). Random intercepts at the chain, insurance, and municipality level, as well as fixed effects were included in the model.Outcome dichotomised based on sensitivity analyses.an individual level logistic regression model was used to evaluate the effect of belonging to a chain of at least five contacts per case on odds of death	models included age, gender and individual comorbidities and regional health system and socioeconomic measures	fatality decreased by 48% (95% CI: 45–51%) among members of chains with five or more contacts per caseon an individual level, odds of dying decreased by 55% (95% CI: 51–58%) among members of contact tracing chains of 5 or more contacts per case compared to those with fewer1.8% of total deaths (830) were estimated to have been prevented by tracing five or more contacts
Fetzer *et al*. [76]	varying exposure across English LTLAs to delays in contact tracing following a database error in which 15 841 positive cases were accidentally missed from data sent to contact tracing teams	new infections *per capita* and new COVID-19 deaths *per capita*	split local authorities based on whether contact tracing was strongly or relatively little affected (above/below median). Performed an analysis of covariate balance. Quantitative estimates made using difference-in-difference regression	controlled for the nonlinear nature of case growth by controlling for previous levels and trends in SARS-CoV-2 spread and, second, for a multiplicity of more than 50 area characteristics. The area characteristics included employment shares in one-digit industries, educational attainment, socio-economic status of the resident population, which also captured shares in full time education or in university, and regular in- and out-commuting flows. These time-invariant measures were interacted with a set of date fixed effects to account for potential nonlinear growth	18.6 (90% CI 10–25) additional infections per late referral to contact tracing reported in the 6-week post-treatment period.0.24 (90% CI 0.06–0.42) new COVID-19 deaths per late referral to contact tracing overall in the following six weeks. (confidence intervals shown graphically so read off using a ruler)
Gianino *et al*. [60]	intensity of contact tracing policies as measured by the OxCGRT	incident cases	vector autoregression modelling with Granger causality testing. Time lags were identified as best fitting to the data as measured by the Akaike information criterion (AIC). Stratified by countries	other interventions measured in the OxCGRT	no convergence in the VAR algorithm for contact tracing in Germany or SpainItaly: *p*-value for the Granger causality test for contact tracing policy = 0.984UK: *p*-value for the Granger causality test for contact tracing policy = 0.984 with a best-fitting lag of 8 days
Kendall *et al*. [65]	implementation of contact tracing app compared to no implementation among English LTLAs. Some of the time period was also comparing a broader NHS Test and Trace intervention, which was implemented more widely (without the contact tracing) partway through the study; after this the difference between LTLAs was the app alone	R (reproduction number), incidence and nowcasting (combined R with incidence *per capita* to provide a simple nowcast)	maximum-likelihood estimation of the growth rate r and corresponding RBack-calculation approach to estimate timing of new infections. Bayesian estimation of the instantaneous reproduction number R. Report posterior mode rather than posterior mean as the central estimate to reduce oversensitivity to the priors when the number of infections was low. Synthetic control created via combining data from all other local authorities and weighting their contributions to match the evolution of R on the Isle of Wight before the NHS Test and Trace launch	synthetic control based on weighted average R of other areas in England matching the area where the intervention was implemented (Isle of Wight) in mean R prior to launch of the intervention and in distributions of age and ethnicity	before and after analysis: Reduction in maximum likelihood estimates of Rt from 1.3 to 0.5. Compared with other local authorities, using pillar 1 data—the Isle of Wight went from having the third highest reproduction number before the Test and Trace programme, to the 12th lowest afterwards. Estimates of R on the Isle of Wight post intervention were lower (95% CI's did not overlap, shown graphically) than those estimates for the rest of England for Pillar 1 (*p* < 0.001) and Pillar 2 (*p* < 0.001) testing data sources and a combined Pillars 1 and 2 testing source (*p* < 0.001)Synthetic control analysis: R was lower for the Isle of Wight than for its synthetic control for most of the period after the Test and Trace launch (difference was significant at the 5% level between May 15 and May 26, lower than the control but not significant at 5% level at other times between May 5 and June 14. Reported graphically.) Synthetic control analysis with additional sociodemographic information: R was lower for the Isle of Wight than for its synthetic control for most of the period after the Test and Trace launch (difference was significant at the 5% level on 15 May, 16 May, 19 May and 20 May, and was mostly lower but not significantly so at the 5% level for the 5 May–June 14 period. Reported graphically)
Vecino-Ortiz*et al*. [73]	proportion of cases detected via contact tracing	mortality from COVID-19	log-log fixed effect models with lags: 21 days (model 1), 28 days (model 2), 42 days (model 3), and 56 days (model 4). Incrementally included lags to assess the effectiveness of contact tracing (sequential model)	fixed effects for departments. Models control for the prevalence of active cases, second-degree polynomials and mobility indices. Robustness checks including supply-side variables such as number of ICU beds and models of random effects design	average of cases identified through contact tracing: 30.91% (range 0% to 100%) total deaths by department: mean 2.65 (range 1 to 24) an increase in 10% in the proportion of cases identified through contact tracing was associated with mortality reductions in the next 21 to 56 days of between 0.8% (*β* = −0.078, s.e. = 0.034, *p* < 0.024) and 3.4% (beta = −0.229, s.e. = 0.041, *p* < 0.001), depending on the model. No substantive differences between the fixed and random effects models were observed. For most models, a quadratic term for the logarithm of proportion of cases that were contact traced was observed. Many models were compared and no findings showed as strong statistical evidence, particularly among models with longer lags
Wymant *et al*. [74]	per cent population who were active contact tracing app users	cumulative cases	four methods: matched neighbours regression (primary); stratified linear regression in clusters; matched pairs regression; matched pairs regression adjusted for manual contact tracing locallystratified into three time periods according to app features and deployment	adjusted for lower tier local authority (LTLA, i.e. small geographical district) urban/rural location, poverty and GDP. LTLAs were stratified into quintiles on the basis of cases during phase 0 and only examined within these strata. Matching and clustering procedures. One model additionally controlling for manual contact tracing performance	matched neighbours regression (primary analysis), per cent reduction in cases for every percentage point increase in app use:Phase 1: 1.09 (95% CI 0.04–2.14) (bootstrap 95% CI 0.15–2.16) Phase 2: 2.66 (95% CI 1.75–3.56) (bootstrap 95% CI 0.80–4.71) Phase 3: 2.26 (95% CI 1.50–3.00) (bootstrap 95% CI 1.60–3.19)
testing strategies					
Chew *et al*. [57]	intensity of contact tracing and testing policies as measured by the OxCGRT	growth rate of cases (growth rate of deaths also considered). Assumes growth rate is [*X(t)* − *X(t − 1)*] × 100*/X*—(0) so all scaled relative to initial value on 2nd May 2020	deep neural network	global mixture of other control interventions as measured by the OxCGRT—the importance of which is extracted by the method. Public sentiment was measured in twitter analyses	at the global scale, contact tracing had a minimal impact on growth rate. Testing was positively associated with growth rate.Asia: contact tracing had the biggest impact on growth rate (−0.3)Africa: contact tracing was positively correlated with growth rate (+0.25)Europe: contact tracing very weak correlationN. America: contact tracing weak and positive, Testing negative (−0.05)S. America: contact tracing weak and negative (−0.02), Testing strong negative (−0.09)For country clusters: contact tracing was the top measure
Gorji *et al*. [20]	enrolled cohorts with greater than one week of asymptomatic testing and daily contact testing, compared to newly enrolled cohorts	7-day average test positivity rate (TPR) and incidence rate ratios (IRR)	the 7-day average TPR was smoothed using a first-order local polynomial regression (LOESS) algorithm with bandwidth 0.65The incidence rate was associated with the 7-day average TPR by normalizing the number of new positive cases by the person-time tested.Incidence rates among repeat testing cohorts and newly enrolled cohorts were compared as the IRR	sensitivity analyses were conducted among 1) only cross-border commuters to assess contamination and 2) within the tourism sector to investigate potential effects of imported cases	positive test results were found among 215/121 364 tests (27 514 individuals)The estimated reduction in the incidence rate among tested cohorts compared to newly enrolled individuals was 18% (95% CI– 25–52%, *p* = 0.36) for the week 1 cohort, 47% (95% CI 5–27%, *p* = 0.03) for the week 2 cohort and 50% (95% CI 4–76%, *p* = 0.04) for the week 3 cohort
Haug *et al*. [64]	increase in score of quarantine policies and enhanced case detection policies (presence/absence), part of ‘Case identification, contact tracing and related measures’ as measured by the CCCL study (Desvars-Larrive A. *et al*.)	Rt—effective reproduction number	four approaches: ‘case-control analysis’*; step function Lasso regression; random forest regression; transformer modelling.* Note cases and controls were defined by exposure not outcome	methods employed varying approaches but included control for (1) epidemic age (days after the country had reached 30 confirmed cases), (2) the value of Rt before intervention type took effect, (3) total population, (4) population density, (5) the total number of NPIs implemented and (6) the number of NPIs implemented in the same category	quarantine:step function Lasso regression: −0.2 (1%) changes in RRF regression: 0.0023 (9%) changes in RTransformer modelling: 0.023 (2%) measure of effect on RtEnhanced case detection:case-control analysis: −0.19 (3%) changesin RStep function Lasso regression: 0 changes in RRandom Forest regression: 0.0032 (9%) changes in RTransformer modelling: −0.106 (2%) measure of effect on Rt
Hong *et al*. [63]	evidence for an interaction between an increase in contact tracing policy (as measured by the OxCGRT) and other classes of NPIs	rate of increase or decrease in the cumulative number of confirmed cases (DRICS) of SARS-CoV-2 from maximum cumulative case increase to 6 days later. To generate this variable, the highest value of the average increase rate of the cumulated number of confirmed cases (IRCs) between the 1 January and the 15 June 2020 was divided by the average cumulative case increase rate 6 days after the record high date for each country, considering the incubation period	linear regression of increased in NPI score on DRICS outcome measure. Contact tracing included as an interaction term for Movement and Assembly restrictions	other NPIs included in the OxCGRT and their interactions with contact tracing: school closures, workplace closures, cancelling public events, gathering size restrictions, public transport closures, stay at home requirements, internal movement restriction, international border restrictions	assembly variable interactions and movement variable interactions, fully adjusted model: interaction between contact tracing and school closures index, *β* = 3.930 (s.e. 1.283), *p* < 0.01 interaction between contact tracing and workplace closures index, *β* = −1.771 (s.e. 0.678) *p* < 0.10Interaction between contact tracing and cancelling public events index, *β* = −3.245 (s.e. 1.156) *p* < 0.01 interaction between contact tracing and gathering size restrictions index, *β* = –0.093 (s.e. 0.594), *p* > 0.1Interaction between contact tracing and public transport closure index, *β* = 0.627 (s.e. 0.534), *p* > 0.1interaction between contact tracing and stay at home requirements index, *β* = 1.176 (s.e. 0.832), *p* > 0.1 interaction between contact tracing and internal movement restrictions index, *β* = −0.436 (s.e. 0.605), *p* > 0.1 interaction between contact tracing and international movement restrictions index, *β* = −0.520 (s.e. 0.605), *p* > 0.1Interpretation of the regression model coefficients was not made explicitly; we assumed the coefficient represents the change in the outcome for a one unit change in the OxCGRT score. Positive values are associated with a more rapid decline from the peak
Leffler *et al*. [66]	*per capita* testing Intensity of contact tracing policies as measured by the OxCGRT	*per capita* COVID-19 mortality, modelled as log per-capita mortality	multivariable linear regression analysis	potential confounding variables were included based on a backwards stepwise procedure. These included weeks spent in lockdown, with international travel restrictions, and using masks. and *per capita* testing levels, were retained in the model. Urbanization, prevalence of obesity, and average ambient temperature were retained in most models	*per capita* testing (log) by 16 April was not negatively associated with per-capita mortality (log) by 9 May (coefficient 0.264, 95% CI: −0.323 to 0.851, *p* = 0.38) *per capita* testing (log) by 4 April was not significantly associated with per-capita mortality (log) by 9 May (coefficient −0.0504, 95% CI: −0.378 to 0.278, *p* = 0.76) There was only weak statistical evidence for an association between contact tracing policy (change in OxCGRT rating from none; limited to complete) with *per-capita* mortality (log) by May 9 (coefficient 0.6674, 95% CI: −0.357 – 0.006, *p* = 0.06)
Pavelka *et al*. [10]	pre- and post-implementation of mass testing rounds	prevalence reduction (1- risk ratio)	impact of mass testing was assessed via the change in test positivity rates in counties with at least two mass testing campaigns. Crude prevalence ratios (cPR) estimated the change in test positivity between mass testing campaigns, including Wald-Normal confidence intervals. Binomial confidence intervals were calculated for the proportion of tests that were found positive. A Bayesian negative binomial regression model was used to explore heterogeneity between counties in the estimated reduction in test positivity in subsequent rounds of mass testing	the number of positive tests in each county was modelled with a pooled county specific intercept, demographic, socioeconomic and ethnic covariates, the number of preceding rounds of mass testing, attendance rates and test positive rates in any previous round of mass testing, the reproduction number leading up to the first round of mass testing as well as the logarithm of the number of tests as an offset variable	a total of 50 466 participants tested positive. The proportion of positive tests was 3.91% (range across counties: 3.12 to 4.84%) in the pilot, 1.01% (range: 0.13 to 3.22%) in round 1, and 0.62% (range: 0.28 to 1.65%) in round 2.The adjusted prevalence reduction was 56% (95% CI: 52 to 59%).The estimated reduction between rounds varied considerably by county but not regionally, from 29% in county Považská Bystrica to 79% in county Medzilaborce
Pozo-Martin*et al*. [70]	testing volumes. Contact tracing as measured using the OxCGRT	time-varying average daily growth rate (wADGR) of the cumulative weekly number rate (wADGR) of the cumulative weekly number of confirmed SARS-CoV-2 cases	two week lag to effect on growth rate. Longitudinal multilevel modelling stratified in two time periods: Jan–Jun 2020 and Oct–Dec 2020.Multivariable linear mixed model for longitudinal data (mLMM) with the probit transformation of the wADGR (probit_wADGR) as the response variable. Maximum Likelihood estimation and Bayesian fitting.Multivariable beta regression generalized linear mixed model (mGLMM) with a probit link function using wADGR as the response variable to estimate average marginal effects (AME) of the NPIs on the wADGR	other concurrent interventions as measured by the OxCGRT. The following country-specific control variables were included: baseline number of cases between the start of the epidemic and the implementation of the first in-country social distancing NPI, weekly temperature, sociodemographic index (SDI), gross domestic product (GDP) *per capita* based on purchasing power parity, percentage of total population living in urban areas, percentage of gross domestic product spent in health, household size, Palma ratio (a measure of income inequality), and democracy index	first time period: contact tracing not retained in model during variable selection proceduresTests per 1000 population: multivariable linear mixed model (95% CI) MLE:− 0.005 (− 0.008, − 0.002); Bayesian (95% CI)—0.004 (−0.008, −0.002); multivariable general linear mixed model rMLE:− 0.004 (− 0.007, − 0.001); AME −0.02.Second time period: contact tracing (not retained in model during variable selection procedures) Testing of anyone showing COVID-19 symptoms: multivariable linear mixed model (95% CI) MLE: 0.17 (0.01, 0.32); Bayesian (95% Cr Intervals) 0.19 (0.03, 0.35); multivariable general linear mixed model rMLE: 0.28 (0.16, 0.39) ; AME 0.89 Open public testing to asymptomatic people: multivariable linear mixed model (95% CI) MLE: 0.13 (− 0.03, 0.30); Bayesian (95% Cr Intervals) 0.13 (− 0.03, 0.30); multivariable general linear mixed model rMLE: 0.26 (0.12, 0.40) ; AME 0.83
Spiliopoulos [71]	contact tracing and testing policies as measured in intensity by the OxCGRT	confirmed cases; deaths. The growth rate of confirmed cases or deaths was calculated as the log difference in the cumulative confirmed cases or deaths for two consecutive days multiplied by 100 (i.e. approximate percentage growth rates)	econometric procedures: structural multi-equation modelling including latent variables capturing the effects of unobservable determinants, to improve upon the external validity. Lags incorporated (14 days longer for deaths than cases)	other interventions measured by OxCGRT. National-level unobservable characteristics were modelled using unique random effects. Fixed effects for the impact of the day of the week on case and death growth rates were included	contact tracing:case growth rates: coefficient = 1.93, *p* = 0.38) Death growth rates: coefficient = 1.31, *p* = 0.52). Testing policy:Case growth rates: coefficient = 66.03, *p* < 0.0001), indicating a decreased case growth with increased testing Death growth rates: coefficient = 42.81, *p* < 0.0001), indicating a decreased death growth with increased testing
Ssentongo *et al*. [72]	testing policy intensity as measured by OxCGRT on a 4 point scale	incident cases	multivariate negative binomial time series model with lags. The conditional mean was split into three components representing endemic (1 component) and epidemic (2 components) transmission, with the strength of connection between countries weighted according to spatial relationships	random effects model fitted to within and between country spread. Accounted for population size, and time-varying stringency of controls and weather	testing (one week ago) had a relative risk of 0.817 (95% CI 0.729–0.918) on the level of *within*-country transmission this weektesting (one week ago) had a relative risk of 2.322 (95% CI 1.676, 3.218) on the level of *between*-country transmission this week
Yalaman *et al*. [75]	each unit increase in the OxCGRT scale for contact tracing (None; Limited: for some cases; Comprehensive: for all cases)	COVID-19 fatality rate as calculated from COVID-19 cases and deaths. Calculated as number of deaths in closed cases, i.e. those who have recovered or died. Overall mortality rates examined as secondary analysis	panel regression with time fixed effects, robust standard errors. Daily time variant measures transformed to biweekly. Total test numbers, stringency and contact tracing variables lagged by two weeks	time fixed effects to control for effect of time. Country-level potential confounders controlled for: number of biweekly tests; stringency score from OxCGRT, number of hospital beds per 1000 population; number of physicians per 1000 population, proportion of population aged greater than 70 years, percentage of smokers, per cent of adult population with diabetes, log population, GDP *per capita*, fiscal stimulus	contact tracing intensity and numbers of biweekly tests were negatively associated with case fatality rates (*β* = −0.16, *p* < 0.10 and *β* = −0.02, *p* < 0.01 respectively), outlier countries included. Statistical evidence for associations with contact tracing was weak. Contact tracing intensity and numbers of biweekly tests were negatively associated with case fatality rates (*β* = −0.13, *p* < 0.05 and *β* = −0.01, *p* < 0.1), outlier countries excluded (countries in the top 5% of case fatality rates in a two week period). Statistical evidence for the association with testing was weak. Contact tracing intensity was negatively associated with overall mortality rates (*β* = −0.4749, *p* < 0.01), outlier countries included. Contact tracing intensity was negatively associated with overall mortality rates (*β* = −0.2962, *p* < 0.01), outlier countries included. There was not statistical evidence for an association between numbers of biweekly tests and mortality rates. Standard errors and 95% CIs were not reported
Zamanzadeh*et al*. [78]	contact tracing and testing policies as measured in intensity by the OxCGRT	ratio of SARS-CoV-2 new infections per 100 000 population	De-biased LASSO regression. Variables normalized to avoid problems with scaling, which also makes all variables continuous, including the contact tracing OxCGRT exposure variable	nine models were run: one including only interventions (business closures, stay-at-home orders, school closures, contact tracing intensity and tests per population); seven sequentially added country socio-economic, governance and healthcare variables and one included all variables. Continent, time and country fixed effects were used in all models	model included other interventions, socioeconomic, governance and healthcare indicators, continent, time and country fixed effects:Each standard deviation increase in contact tracing policy was associated with a −0.1008 *decrease* in the standard deviation of numbers of new infections per 100 000 population (reported as showing statistical significance at 99 per cent of credible intervals). Each standard deviation increase in numbers of tests per 100 000 population was associated with a 0.0039 *increase* in the standard deviation of numbers of new infections per 100 000 population (reported as showing statistical significance at 99% of credible intervals)
Zhang *et al*. [77]	Liverpool City local geographical areas (MSOA) receiving the rapid asymptomatic testing intervention Nov 6 2020–2 Jan 2021	hospitalizations of COVID-19 cases (confirmed or clinically diagnosed according to Hospital Episode Statistics)	synthetic control method, in which an untreated version of the intervention MSOAs was generated for the time period under study using a weighted combination of areas not exposed to the intervention and the intervention was then estimated comparing the trend in the outcome between the intervention areas and the synthetic control. Assumed a time lag of two weeks so outcome data were taken from 19 Nov 2020	control of confounding occurred via selection of characteristics on which to select MSOAs used in creating the synthetic control. This was done based on MSOA socioeconomic and demographic characteristics, PCR testing rates and on pre-intervention COVID-19 hospitalization rates and follow-up time limited to that in which asymptomatic testing was still higher in Liverpool than elsewhere in the country, where it was later rolled out. The effect of other epidemic controls, namely England's ‘Tier’ epidemic control system was accounted for by adjusting upwards hospitalizations in MSOAs moving from Tier 2 to 3 in the follow-up time, with this approach varied in sensitivity analyses	for 6 Nov–3 Dec 2020 period, no assumed reduction in admissions related to being in Tier 3 compared to Tier 2: −43% (−57% to −29%) lower hospitalizations in intervention MSOAs compared to synthetic control, difference of −146 hospitalizations in absolute terms (−192 to −96), *p* < 0.001 For 6 Nov 2020 to 2 Jan 2021, no assumed reduction in cases for being in Tier 3 compared to Tier 2, −16% difference in hospitalizations in intervention MSOAs compared to synthetic control (−27 to 0), −133 hospitalizations (−239 to −3) in absolute terms, *p* = 0.07.For 6 Nov 2020 to 2 Jan 2021, assuming a 17% reduction in hospitalizations due to being in Tier 3 compared to Tier 2, difference of −25% (−35% to −11%) in intervention MSOAs compared to synthetic control, −239 in absolute terms (−333 to −104), *p* < 0.001
isolation					
Lopez *et al*. [61]	cases isolating in hotels versus those isolating at home	number of secondary household (HH) cases	multivariate logistic regression	potentially confounding characteristics included in the final model, selected via stepwise procedures, included household member's sex and age and overcrowding index	*N* = 229 contacts, *N* secondary cases = 123Hotel 69/139 secondary HH casesHome-isolated: 54/90 secondary HH casesadjusted OR = 1.67 (95% CI 0.89–3.12) for infection among household members of home versus hotel isolated patients for home compared to hotel isolation
Love *et al*. [67]	intervention compared to control groupIntervention: Daily contact testing: daily contact testing with seven self-administered LFD tests, with release for 24 h based on a negative LFD result Control: Standard isolation group: a single self-taken PCR swab and self-isolation for 10 days	attack rate: percentage of secondary contacts (close contacts of SARS-CoV-2-positive study participants) who became COVID-19 cases (tertiary cases) in each group	modified intention-to-treat analysis excluding those who actively withdrew from the study as data from these participants were no longer held. Bernoulli regression models using Huber-White (robust) sandwich estimator clustered standard errors were used to account for the inflation due to participants being grouped within households. Interactions were tested for significance by Wald tests with a significance level of 0·05. It was a non-inferiority study (margin of 1.9%)	intervention was randomized. Adjusted models also included household exposure, vaccination status and ability to work from home	number of tertiary cases (close contacts of SARS-CoV-2-positive study participants who became COVID-19 cases): self-isolation 393; DCT 325. Number of tertiary cases per secondary case: self-isolation 0.14; DCT 0.16 Adjusted attack rates among secondary cases (%). Self-isolation 7.5% (95% CI 6.7, 8.3%); DCT 6.3% (95% CI 5.6, 7.0%). Difference (DCT versus self-isolation) −1.2% (95% CI −2.3, −0.2%)
Malheiro *et al*. [68]	intervention and control cohorts were defined based on whether cases were subjected to contact tracing and quarantine measures before the laboratory confirmation of disease	median number of secondary cases per index case; proportion of cases with secondary cases	χ^2^ and Mann–Whitney *U* tests were used to evaluate the distributions of categorical and continuous variables, respectively, between the intervention and control groups. Stratification by time period	stratification according to whether a case occurred before or after national lockdown plus a lag equal to one incubation period	222 cases were identified pre-lockdown (27 intervention) and 329 post lockdown (71 intervention) Median number of secondary cases by index case was lower for the cases reported under the state of emergency at a 7-day lag (median: 0, IQR: 0–0, in both time periods, *p*-values were 0.807 and 0.912 pre and post lockdown). Pre-lockdown 11.1% of intervention cases had a secondary case compared to 21.5% among controls cases (*p* = 0.314) Post-lockdown 14.1% of intervention cases had a secondary case compared to 14.0% among control cases (*p* = 1)
Nam *et al*. [69]	centralized isolation of all confirmed cases (varying locations). Compared to non-centralized isolation (synthetic controls constructed based on Sweden)	per cent decreasing new cases. The absolute effect average was defined as the average per cent decrease rate of the trend after intervention/day. The absolute effect cumulative was determined as the sum of the absolute effect average. The relative effect average was the actual percentage that decreased after the intervention, and the posterior probability of a causal effect is the repeatability of causality	synthetic control analysis using the Causal Impact software package. Given the intervention (countries with centralized case isolation) and control time series (Sweden, with cases adjusted upwards in relation to regional neighbours to adjust for perceived low testing rates), a Bayesian structural time series model was used to reconstruct a counterfactual to which the intervention could be compared	interventions were only assessed when they were implemented at least 13 days apart (results only shown in these instances). Otherwise their effects were not examined as independent. Other interventions examined included closure of schools, closure of public areas, closure of cities and closure of borders	Chinapre-intervention 17.29 (95% CI 12.90–22.04); post-intervention 0.72 (95% CI 0.52–0.96)absolute effect average: 16 (95% CI 10–23); relative effect average: 96 (95% CI 60–132) posterior probability of a causal effect was 99.995, *p* < 0.0001. When centralized isolation of confirmed cases was added, closure of schools, public spaces, cities and borders were already enforcedJapanpre-intervention 12.60 (95% CI 8.30, 17.27); post-intervention 7.76 (95% CI 6.00, 9.58) absolute effect average: 5.8 (95% CI −1.2, 13); relative effect average: 43 (95% CI −8.7, 94) posterior probability of a causal effect was 95.085, *p* < 0.05. No other interventions in force
TTI as part of a package					
Chan *et al*. [56]	implementation periods of case identification, contact tracing and related measures compared to periods without their implementation	*reductions in Rt.* Rt was estimated from reported case numbers using deconvolution methods and Japanese delay distributions	ratio of Rt before and after the implementation of intervention portfolios. *Aggregate intervention effectiveness in reducing Rt* was calculated as follows: *Portfolio effectiveness* was defined as the ratio of the estimated reproduction numbers at the time of initiation of the portfolio and when the specific portfolio combination ended. *Intervention effectiveness* for the period over which the portfolio was enacted was the portfolio effectiveness divided by the number of interventions being implemented. *Aggregated intervention effectiveness* is the product, for each country, of the individual intervention effectiveness estimates over the time period of the study	from one intervention portfolio implementation to another (on any subsequent day) the previously calculated portfolio effectiveness was kept constant (also in consideration of the absence of information about the ending of interventions after implementation)	median aggregate intervention effectiveness across countries = 10^0.05^ = 1.12. A ratio > 1 indicated effectiveness of the intervention in reducing Rt. 25th percentile = 100̂ = 1 and 75th percentile = 100̂.1 = 1.26 (median provided as text but the variation was only presented graphically and extracted from the figure)
Heo *et al*. [62]	average measure in the OxCGRT across 3 areas	daily case numbers	segmented Poisson model. Calendar time breakpoints: 17 Feb (1st case related to Daegu church service); 6 May (first case related to nightclub outbreak); 10 Aug (first Monday after peak summer vacation week); 20 Oct first stage social distancing relaxation. Lagging from 0 to 10 days. Crude (univariate) analyses and analyses with all indices in the model conducted. Seoul and non-Seoul metropolitan areas strata considered. Results reported for each of 0–10 days’ lags	other policies on the given day, in South Korea, as documented according to the OxCGRT and the South Korean government social distancing index: restriction on gatherings, stay at home requirements, domestic travel restrictions, school closing, workplace closing, cancelling of public events, international travel controls, income support, debt/contract relief, public information campaign, testing policy	coefficients indicate that for a one-unit change in the control index (all normalized to a scale of 0–100), the difference in the logs of expected counts (COVID-19 daily confirmed cases) given the other indices in the model held constant. Negative coefficients indicate transmission reduction. No measures of statistical uncertainty given. Seoul metro, adjusted for other indices, (no lagging, 10 days lagging): (no lagging to 10 days lagging): values ranging between *β* = −0.1193 and *β* = −0.0286Non-Seoul metro, adjusted for other indices, (no lagging to 10 days lagging): values ranging between *β* = −0.7129 and *β* = 0.1895 Whole country, adjusted for other indices, (no lagging to 10 days lagging): values ranging between *β* = −0.1015 and *β* = 0.1057

#### (i) Contact tracing (seven papers)

**Figure 3.**
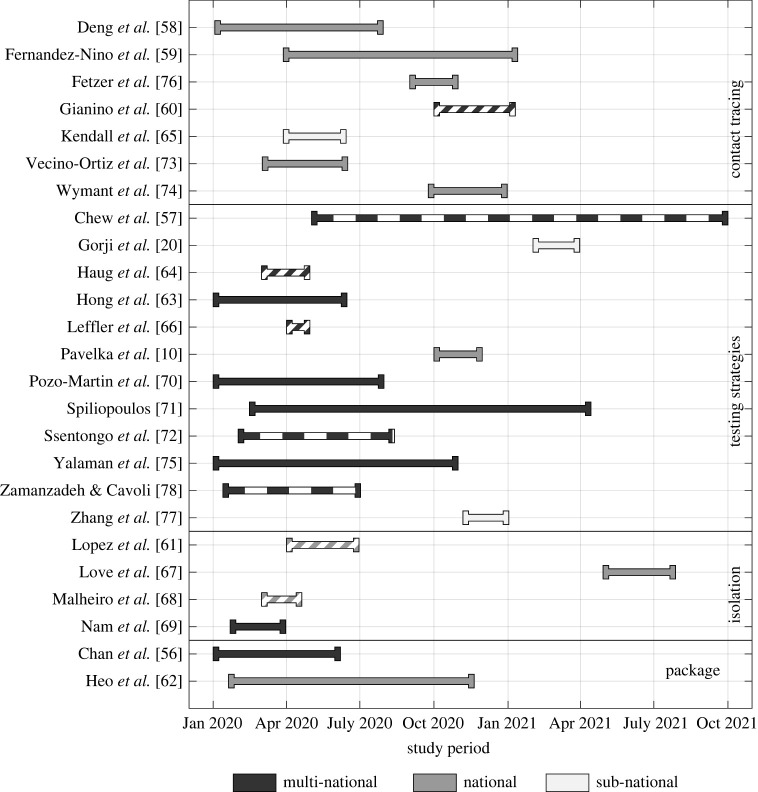
Graphical representation of the 25 studies showing the associated time interval of the study (*x*-axis), the scale of interventions and analysis (multi-national black; national dark grey; sub-national light grey) and the reported statistical significance of the results: solid bars are studies where there was a significant reduction in transmission, cases or deaths due to a TTI intervention; diagonal hashed bars are where the results were not statistically significant; and long dashed bars are where findings were conflicted in different regions, times or scales.

Seven papers considered the impact of contact tracing (as part of a TTI scheme) on the dynamics of the pandemic, figure 3. Six of these studies considered data from single countries and made use of spatial or temporal heterogeneity in contact tracing to assess its population impact [58,59,65,73,74,76]. One study considered contact tracing policies across multiple countries [60].

Deng *et al*. [58] showed that across 313 Chinese cities from January to July 2020, the number of new COVID-19 cases were significantly negatively correlated with the stringency of contact tracing at lags of 0, 7 and 14 days.

Fernandez-Nino *et al*. [59] and Vecino-Ortiz *et al*. [73] both examined contact tracing in Colombia. Vecino-Ortiz *et al.* considered the proportion of cases identified through contact tracing between March and July 2020 as a measure of the intensity of contact tracing, and found that the number of deaths was negatively correlated with the intensity of contact tracing 21 days previously, with some evidence that the effect on deaths for a given contact tracing intensity tapered off somewhat as tracing effort increased (a possible ‘saturating' effect). Fernandez-Nino *et al.,* considering data between March 2020 and January 2021, observed that cases that were part of chains of at least five contacts (and hence when contact tracing was more comprehensive) had a statistically significant 48% reduction in fatality.

Three papers focused on data from the UK; Kendall *et al*. [65] and Wymant *et al*. [74] considered the NHS COVID-19 contact tracing app in England and Wales, while Fetzer *et al*. [76] concentrated on delays to tracing. Kendall *et al*. [65] focused on the pilot of the contact tracing app launched on the Isle of Wight during May and June 2020, and estimated the reproductive ratio *R_t_* on the Isle of Wight and in 150 other Upper-Tier Local Authorities in England. It was found that on the Isle of Wight, *R_t_* had reduced from 1.3 (the third highest in England) before the app to 0.5 (the twelfth lowest) after the piloting of the app. Wymant *et al*. [74] supported Kendall *et al*.'s [65] findings; looking at the proportion of the population that used the app in each of 338 Lower-Tier Local Authorities they found a 1.1% (between October and November 2020) and 2.7% (between November and December 2020) reduction in cases for every percentage increase in app usage. Fetzer *et al*. [76] examined data from September 2020, when data handling issues within the public health authority led to delays of 6–14 days in the time taken to perform contact tracing; compared to such long delays, regression analysis suggested that timely tracing led to 63% fewer infections and 63% fewer deaths over this period.

By contrast, Gianino *et al*. [60] analysed data from Italy, Germany, Spain and the UK between October 2020 and January 2021, and could find no significant correlation between contact tracing and the incidence of SARS-CoV-2 cases. In its approach and use of the OxCGRT [83], which provides a three-level categorization of contact tracing intensity, the study by Gianino *et al*. [60] has more in common with the statistical approaches examined below in studies exploring testing strategies.

Overall, six of the seven studies assessing contact tracing found that contact tracing (in addition to other controls in use at the time) led to a significant decline in transmission; all of these studies relied on detailed information about contact tracing strength at an individual or small spatial scale. The only paper not finding a significant relationship [60] used coarse-grained data at the country scale from the OxCGRT [83].

#### (ii) Testing strategies (12 papers)

Nine papers performed statistical analyses across multiple countries to assess the impact of changing patterns of control [57,63,64,66,70–72,75,78], while three examined testing strategies in single countries [10,20,77]. Many of the cross-country studies used the OxCGRT [83] to inform the type and strength of epidemic controls in each country over time. The studies varied in the number of countries/regions examined, the time scale over which the analysis was performed, and the measure used to assess the impact of testing and contact tracing using early data (up to May 2020), those that used later data when assessing contact tracing and then those that consider the strength of community testing (separate to any measures of contact tracing).

The earliest pandemic data (up to May 2020) showed the smallest impacts of testing and/or contact tracing. Haug *et al*.'s [64] analysis of 226 countries during March and April 2020 concluded that contact tracing was one of the least effective interventions as measured by a change in the reproductive ratio. Leffler *et al.* [66] analysed data from five countries (China, Macau, Cameroon, Sierra Leone and Sudan) in April and May 2020 and found only weak statistical evidence for an association between contact tracing completeness and lower *per capita* mortality (*p* = 0.06). Across the early epidemic period of 37 OECD countries, Pozo-Martin *et al*. [70] found no statistical evidence for an association between contact tracing completeness and average daily case growth rate.

Later results from cross-country statistical analyses tend to support a positive impact of contact tracing intensity (together with testing) on transmission. Hong *et al*. [63] studied 108 countries from January to June 2020 and found in an unadjusted analysis that contact tracing was significantly correlated with a more rapid decline from peak cases. However, adjusting for concurrent interventions, and treating contact tracing as an effect modifier of these interventions, the role of contact tracing was more mixed (contact tracing and school closures led to a significantly faster decline, but contact tracing combined with cancelling public events or workplace closures led to a significantly slower decline). Yalaman *et al*. [75] compared 138 countries over the period January to October 2020, and found that contact tracing had a strong and significant association with reduced population level mortality rates. Examining the 100 days after the first case was reported among 37 low- and middle-income countries, ranging from January to July 2020, Zamanzadeh and Cavoli [78] found that increased contact tracing intensity (measured using the OxCGRT scale [83]) was associated with lower incidence. Chew *et al*. [57], using neural networks to assess the impact of testing and contact tracing on growth rates between May 2020 and October 2021, found that while the results at a global and continental scale were mixed, contact tracing was the top measure for reducing growth rate when countries were clustered according to their dynamics. Although, Spiliopoulos [71] did not find evidence for an association between contact tracing and either case or death growth rates using data from February 2020 to April 2021.

Seven papers considered the impact of testing separately or independently to any available measures of contact tracing. Both positive and negative impacts of testing on transmission were reported; this may be due to conflicting actions of testing; more testing leads to a higher proportion of infections being detected but if cases are effectively isolated this should lead to fewer subsequent infections. Spiliopoulos [71] showed that testing had a significant negative impact on the growth rate of cases and deaths based on the data from 132 countries between February 2020 and April 2021 and the strength of this impact increased with the strength of the testing. However, studying the early epidemic period of LMICs, Zamanzadeh and Cavoli [78] found that increased testing was associated with higher cases, though they were not able to separate out effects on transmission from the effects of enhanced case detection on the proportion of cases identified. Leffler *et al*. [66] found no statistical evidence for an association with log per capita testing and per capita mortality across five countries from April to May 2020. Pozo-Martin *et al*. [70] found varying results depending on study period across 37 countries: from January to September 2020 they found that for every test per 1000 people there was a 0.02% reduction in the weekly growth rate, but later data (October to December 2020) showed that testing was positively correlated with the growth in confirmed cases, possibly indicating a difficulty with case growth rate being an indicator of both case detection and transmission. Ssentongo *et al*. [72] restricted their analysis to 46 countries in mainland Africa using data between January and August 2020 and found that testing (one week previous) significantly reduced the level of within-country transmission by 18%, but was associated with an increase in between-country transmission. Two studies failed to find statistical evidence for an effect of testing on transmission (Yalaman *et al*. [75] and Chew *et al*. [57]).

Studies using later (post-May 2020) and therefore more data, tended to find stronger relationships, with both testing levels and contact tracing leading to better public health outcomes, although this was not universal. However, these statistical analyses highlight the difficulties of assessing control measures by comparing across countries and times. By virtue of incorporating data from many countries, and including many different types of mitigation, the classification of control intensity is necessarily coarse; data from OxCGRT [83] for contact tracing has just three classifications: no contact tracing; limited contact tracing, not done for all cases, and comprehensive contact tracing, done for all identified cases. As shown by the study of Wymant *et al*. [74], heterogeneities exist at relatively small scales and can be informative for understanding the impacts of control. Both testing and contact tracing are likely to lead to an increased proportion of cases that are detected, making it difficult to discern effects on underlying incidence. In addition, such statistical analyses may not be able to fully capture the complex non-linear interactions between epidemiological dynamics, synergies and dependencies between testing and contact tracing, and with other interventions, and behavioural responses.

Three other studies provide more detailed within-country analyses of the impact of testing strategies. Pavelka *et al*. [10] examined the imposition of rounds of mass testing to exit lockdowns in October and November 2020 in Slovakia. In total, over 4 million tests were performed (out of a total population size of 4.5 million adults), with 50 thousand testing positive; while the impact was spatially and temporally heterogeneous, it was estimated that mass testing led to a 56% reduction in prevalence of infection between the first and second rounds. Gorji *et al*. [20] analysed the impact of weekly testing of employees in the Canton Grisons area of Switzerland during February and March 2021; companies enrolled in the testing scheme saw an 18%, 47% and 50% reduction in incidence in employees across three cohorts (with the latter two values being significant at *p* < 0.05) for those that had been enrolled for one, two or three weeks compared to newly enrolled companies. The Gorji *et al*. [20] study echoes similar findings where regular testing has been used on specific sectors of the population including school children and healthcare workers. Zhang *et al*. [77] estimated the effects of an asymptomatic testing pilot using rapid antigen tests on transmission, comparing hospitalizations in Liverpool city middle layer super output areas (MSOA, small geographical areas of around 7200 people) to a synthetic control based on comparative MSOAs without availability of asymptomatic testing in November and December 2020. While the intervention evolved somewhat in its design over time and also included effects of increased publicity around COVID-19 associated with the intervention, the study's primary finding was a reduction in hospitalizations of 25% (35% to 11%) in intervention districts compared to synthetic control.

Taken together, these three studies [10,20,77] show the benefits of large-scale testing of the population—especially when infection is relatively common, as in Pavelka *et al*. [10] and Zhang *et al*. [77]. Earlier in the outbreak, when resources were generally more limited, the actions of testing and contact tracing might have been swamped by more stringent control measures such as lockdowns or social distancing.

#### (iii) Isolation strategies (four papers)

Four publications considered the effects of isolation of (suspected) cases or contacts but considered different aspects of the isolation procedure.

Malheiro *et al*. [68] considered the time between being identified as a contact of a confirmed case and the subsequent laboratory results, with the intervention group being quarantined while waiting for the laboratory results. This study conducted in Portugal from 1 March to 20 April 2020, when there were a range of other strict mitigation measures in place, found slightly fewer traced individuals having secondary cases from the intervention group (13.3% when compared with 17.2%) but the result was not statistically significant and the study included relatively small sample sizes (98 in the intervention group and 453 in the control group).

Lopez *et al*. [61] and Nam *et al*. [69] considered the location of quarantine, with Lopez *et al*. [61] examining hotel quarantine in Spain during April to June 2020 and Nam *et al*. [69] examining the step change in transmission on the introduction of centralized isolation in Japan and China. Lopez *et al*. [61] report more secondary household contacts with household-based quarantine than with hotel-based quarantine (with an odds ratio of 1.67), but the finding was not statistically significant and included only 229 individuals in the study. By contrast, Nam *et al*. [69] took a cross-country approach and report a 43% drop in transmission in Japan and a 96% drop in transmission in China when confirmed cases were centrally isolated, however given the number of other changes occurring in February 2020 (including increased public awareness of the pandemic) any assumptions around causality in statistical analysis should be made with caution.

Finally, Love *et al*. [67] conducted a non-inferiority RCT on around 55 000 adults identified by contact tracing in England between 29 April and 28 July 2021. This study compared home-based self-isolation of contacts for 10 days (policy at the time) versus daily LFD testing for 7 days and no isolation while the LFD tests were negative. The outcome was the proportion of contacts-of-contacts reporting positive tests to the national testing and contact tracing authority, a proxy for the attack rate among this group of contacts. Individuals from each group that became infected had a similar number of contacts (approx. 2.2 per case), and the percentage of them reporting positive tests was lower in the intervention arm (–1.2%, 95%CI of −2.3% to −0.2%) so the daily contact testing intervention was judged non-inferior to home isolation.

Taken together, these studies suggest some uncertainty around the benefit of rapid isolation of contacts (before laboratory results are available [68]) away from the home environment [61,69], with studies either small or unable to disentangle the effects of different concurrent interventions. The findings of the study by Love *et al*. [67] that daily testing is at least equivalent to quarantine in terms of transmission are important, given that it allows substantially more social and economic freedom to those affected.

#### (iv) TTI as part of a broader package of measures (two papers)

Two papers analysed the impact of testing and contact tracing as part of a wider package of measures, so the effects of TTI alone cannot be assessed. Closely aligned with the eight population level studies of testing strategies detailed above, Chan *et al*. [56] considered data from 50 countries and eight suites of control measures from the start of the pandemic to July 2020; a combined package of case identification, contact tracing and related measures led to a net decline in the reproductive ratio but the results were heterogeneous between countries and not statistically significant. Heo *et al*. [62] analysed controls and daily new cases of SARS-CoV-2 in South Korean data from 20 January to 20 November 2020, and considered testing, contact tracing and public information campaigns as a combined control measure as they are tightly clustered in their implementation. The combined control measures were negatively correlated with cases at a number of lags (from 0 to 10 days) but the statistical significance of these correlations is not given, making the findings difficult to interpret.

## 4. Discussion

In total, we identified 417 peer-reviewed studies that examined the impact of testing and/or contact tracing and isolation on the transmission dynamics of infection. The overwhelming majority of these were transmission model-based studies, with only 25 empirical studies meeting our criteria of reporting the real-world impact of testing and/or contact tracing together with some adjustments for confounding factors such as changes to other control measures or population characteristics. Of these 25 studies, 11 adopted a broad statistical approach and attempted to link coarse classification of control measures in multiple countries to their epidemiological dynamics [56,57,60,63,64,66,70–72,75,78]; five considered detailed contact tracing data from either England [65,74,76] or Colombia [59,73]; four considered strategies for isolation after testing or notification as contacts [61,67–69]; two considered within-country stringency of TTI-type controls in China [58] and South Korea [62]; with other papers focusing on the impact of mass-testing [10,77] and weekly testing of people without symptoms [20].

In general, these 25 studies showed that testing and/or contact tracing were associated with reductions in transmission—either measured from the growth of cases, the number of cases *per capita* or the level of mortality in the population. Three of the data analysis studies comparing between country dynamics using data from the earlest stages of the pandemic did not find statistically significant relationships [56,64,66]; this may reflect the strength of other control measures (such as lockdowns) masking the impact of TTI. However, one study focusing on the first 100 days in low- and middle-income countries did find associations between increased intensity of contact tracing and reduced numbers of new infections [78]. Some of the cohort studies [61,68] were considered to have been under-powered to yield significant results. All other studies identified a public health benefit of TTI interventions. Given the variety of ways in which both testing and contact tracing are characterized in different studies, and the different measures adopted for quantifying transmission reduction, it is not possible to pull a coherent metric from the studies for the impact of testing and related controls. There is a clear need for the relationship between TTI and transmission to be framed in a consistent quantitative manner that makes intuitive sense to policy-makers and public health officials. We would highlight Wymant *et al*. [74] as a good example of intuitive quantification which stated that a 1% increase in contact-tracing app usage was associated with a 2.7% reduction in cases (between November and December 2020).

We found a lack of RCTs of TTI interventions, identifying only one in the general population [67]. There are a limited number of other randomized studies but these were in specific settings, and therefore fell outside the scope of our review [84,85]. One was a trial of daily contact testing in schools in England which also found this approach to be non-inferior to contact isolation, consistent with the later findings among the general population [67]. The two experimental studies of concert event attendance with pre-testing compared to no attendance did not specifically measure the benefits of testing [86,87] and so were not included in our review. Both found no statistically significant difference in infection incidence between event attenders and non-attenders post event, though other measures, such as ventilation, face mask mandates, sanitizing and social distancing were also used at the events, and attributing effectiveness to pre-event testing alone is not possible.

The other 24 studies we identified were generally retrospective analyses of data, relying on heterogeneities between cohorts, locations, or times to infer the impact of testing, tracing and isolation methods. The difficulty with such retrospective observational analyses is the role of confounders. These confounders include underlying changes in the incidence of infection, changes to type or intensity of other controls being implemented, changes in the dominant variant, and changes to the public reaction and hence the compliance with mitigation measures. Additionally, statistical analyses, particularly across many diverse countries, might struggle to account for complex nonlinear interactions between epidemiological dynamics, control interventions and diverse behavioural responses; and it may be difficult to provide a consistent measure for the strength of TTI controls. TTI interventions have the additional complication for evaluation that a more efficient testing and tracing scheme will (initially) identify more cases, even though it should reduce the number of infectious individuals in the community.

Our review sought to use a sensitive search strategy with robust screening, review, and appraisal methods to identify the empirical real-world effectiveness of a variety of TTI interventions on SARS-CoV-2 transmission reduction. Our findings make an important and unique contribution to the evidence base; we did not identify previous reviews covering this range of testing, contact tracing and isolation interventions on SARS-CoV-2 transmission outcomes. Two recently published systematic reviews of contact tracing effectiveness, one of infectious diseases more broadly [48] and one specific to COVID-19 [47], did not review testing or isolation strategies. With regards to contact tracing, our review came to similar conclusions as the review of contact tracing for COVID-19 [47] which found a large number of mathematical modelling and simulation studies that predicted the high theoretical effectiveness of contact tracing strategies, alongside a relatively small set of empirical studies, with more mixed results. This difference could indicate implementation challenges with achieving effectiveness from TTI interventions in practice and/or a lack of empirical studies, though the highest quality studies included in both our reviews did find strong evidence of TTI effectiveness on reducing transmission.

Our review has some limitations. Given the large number of search results, it was only feasible to use two reviewers to screen 20% rather than 100% of papers—although the 20% found high levels of agreement between reviewers. While we might have identified more evidence by including preprint papers, this would have risked including lower quality studies or studies that never go on to be peer-reviewed. Future extensions of this review could include key secondary outcomes of TTI interventions hypothesized to facilitate or be proxies for effectiveness, such as proportions of cases identified as contacts, proportion identified prior to symptoms, and testing and tracing delays which could give further insight to the relative effectiveness of different approaches. A number of modelling studies have also considered such fine-grained implementation questions, although generally lack the real-world data to support the policy choices [33]. Synthesis and analysis of evidence relating to barriers to uptake and adherence to TTI measures and effects of social and health inequalities will also be important to inform future intervention development and deployment.

Our review did not consider the costs, either financial to the individuals concerned or to the wider economy nor the potential health, well-being, social and educational harms of different TTI strategies. This is clearly an important and generally overlooked area, as decisions about which control policies to adopt must reflect both costs and benefits. Such a cost-benefit view would also require a more detailed examination of testing using PCR rather than LFD, as PCR tests are considerably more expensive.

While this review sought to identify independent effects of TTI interventions to assess their optimal design and contribution to epidemic control, it is challenging to view each epidemic control intervention as isolated from the others. At the very least, the action of initiating a change in controls may alter the public perception of risks and therefore their level of compliance; the role of enhanced publicity and communication around COVID-19 might have played a role in the effects attributed to contact tracing and asymptomatic testing in this review's included studies, as explicitly mentioned by study authors [65,77]. In addition, important synergies also likely exist. Some countries in Oceania and East Asia used very stringent international border controls to prevent importation, along with extensive contact tracing and physical distancing (when cases were identified) with the aim of local containment and elimination prior to mass vaccination. Of these, Australia and New Zealand are key examples of where combinations of NPIs kept infection to low levels until herd immunity could be gained through vaccination [88,89]. The limited case numbers associated with stringent border controls and other methods are likely to facilitate more effective contact tracing, as the available resources can be focused on far fewer cases [90]. These contexts were very different to countries like the UK in which TTI measures were implemented in a context of varying and sometimes high prevalence. To prepare for future pandemics, these synergies, for which we lack extensive robust empirical evidence, will need to be considered.

There are some clear recommendations that come from this review. First is the need for more robustly designed experimental studies to inform TTI design; such studies need to quantity TTI impact across diverse populations, over different levels of compliance, over different time periods and for different epidemiological characteristics. Second is the need for in-depth studies that can quantify the costs and benefits of different testing and control strategies, and how these might vary between different epidemic phases and between different sectors of society. Finally, there is a need for a better quantification of the interaction between different control measures, which may act either synergistically or may weaken the effect of each other. Associated with all these is the need for a consistent method of reporting findings, such that the results of multiple studies can be compared.

Despite these gaps in our knowledge, two important conclusions can be drawn from the studies we have considered. Firstly, while evidence is imperfect, the majority of studies (including those of highest quality) suggest a considerable impact of testing, followed by isolation and treatment of detected individuals. Pavelka *et al*. [10] highlights the substantial reduction in infection following rounds of nationwide mass testing, which removed a large proportion of infected individuals from circulation within the general population. Similarly, Gorji *et al*. [20] show that regular testing within groups of co-workers can reduce the levels of infection, demonstrating both the utility of testing and the proportion of transmission that occurs in the work environment. Increased access to testing for asymptomatic individuals was also found to have reduced hospitalizations in Liverpool in the UK in late 2020 [77]. While the study by Love *et al*. [67] shows that regular daily testing is non-inferior to isolation of contacts, again emphasizing the strength of testing at detecting cases. Secondly, the studies from the UK [65,74,76] and Colombia [59,73] highlight the benefits of contact tracing as a method of identifying potential secondary cases. These studies of detailed data at a fine spatial scale demonstrate that timely contact tracing in the UK reduced the population-level growth rate or levels of infection; while the analysis of data from Colombia showed how contact tracing reduced mortality, both among infected cases who were identified sooner through contact tracing and at the population scale due to reduced transmission.

We conclude that testing, contact tracing and facilitated isolation can substantially reduce the transmission of infection and improve public health outcomes, and therefore is a key public health tool against future outbreaks—especially before infection-specific pharmaceutical interventions are widely available. However, many aspects of TTI efficacy and real-world effectiveness were unknown at the beginning of the COVID-19 pandemic, and many remain unquantified in general. Considerably more research is required to fully elucidate the epidemiological consequences of TTI under different scenarios (for example, the interaction with variants, vaccination and other controls), as well as the broader costs and benefits of different approaches to TTI.

## Data Availability

The data are provided in electronic supplementary material [91].
